# Novel Functionalized Selenium Nanoparticles for Enhanced Anti-Hepatocarcinoma Activity In vitro

**DOI:** 10.1186/s11671-015-1051-8

**Published:** 2015-09-03

**Authors:** Yu Xia, Pengtao You, Fangfang Xu, Jing Liu, Feiyue Xing

**Affiliations:** 1 Department of Immunobiology, Institute of Tissue Transplantation and Immunology, Jinan University, Guangzhou, 510632 People’s Republic of China; 2 Department of Stomatology, Jinan University, Guangzhou, 510632 People’s Republic of China

**Keywords:** Selenium nanoparticle, Anisomycin, Apoptosis, Anticancer

## Abstract

Selenium nanoparticles loaded with an anticancer molecule offer a new strategy for cancer treatment. In the current study, anisomycin-loaded functionalized selenium nanoparticles (SeNPs@Am) have been made by conjugating anisomycin to the surface of selenium nanoparticles to improve anticancer efficacy. The prepared nanoparticles were fully characterized by transmission electronic microscopy, energy dispersive X-ray spectroscopy, Fourier-transformed infrared spectroscopy, and X-ray photoelectron spectroscopy. The results showed that anisomycin was successfully conjugated with selenium nanoparticles. The size of particles could be effectively regulated through altering the reaction concentrations of sodium selenite and anisomycin. The SeNPs@Am particles (56 nm) exhibited the greatest capacity for cellular uptake. The further study showed that SeNPs@Am entered human hepatocellular carcinoma HepG2 cells in a dose or time-dependent manner via macropinocytosis and clathrin-mediated endocytosis pathways. SeNPs@Am significantly inhibited HepG2 cell proliferation with the low cytotoxicity against normal cells, and dramatically precluded the aggression and migration of HepG2 cells. It also arrested the cell cycle progression at the G0/G1 phase through the activation of the cyclin-dependent kinase inhibitors with inhibition of CDK-2 and ICBP90, and induced the cell apoptosis through activating the caspase cascade signaling in HepG2 cells, markedly superior to anisomycin alone. The findings indicate that SeNPs@Am may be a promising drug for hepatocellular carcinoma.

**Electronic supplementary material:**

The online version of this article (doi:10.1186/s11671-015-1051-8) contains supplementary material, which is available to authorized users.

## Background

Hepatocellular carcinoma (HCC) is one of the most common malignancies worldwide [1]. Owing to its high metastatic potential and resistance to traditional drugs, efficient chemotherapy has become one of the great challenges in clinical treatment [2]. The traditional chemotherapy is usually associated with several shortcomings, such as nonselective distribution of drugs, drug toxicity, and undesired side effects [3]. In addition, most of current anticancer agents usually have short circulation half life and poor aqueous solubility, which hampers therapeutic efficacy of chemotherapy [4, 5]. Thus, new strategies to improve treatment are urgently required. Application of bionanomaterials in the biomedical field has the potential to solve these problems [6]. Nanoparticles (NPs) used as drug delivery systems offer a novel approach for delivery of small chemotherapeutic molecules due to their pharmacokinetics and biodistribution behaviors [7, 8]. NPs as delivery carriers of anticancer drugs have enormous merits, including site-specific targeting [9], reducing doses, ensuring drug efficacy, minimizing side effects, protecting drugs against degradation, and enhancing drug stability [10, 11]. Thus, nanoparticles for drug delivery have gradually been developed as new strategies for cancer therapy [12, 13].

Selenium (Se), an essential trace element, is one of the commonly studied materials in cancer therapy [14, 15]. A substantial amount of evidence has suggested that chemical structures are important determinants of chemopreventive activities of selenium compounds [16]. Novel Se nanoparticles (SeNPs) are attracting increasing attention as potential drug carriers due to their excellent biological activities [17].

Anisomycin (Am), an antibiotic isolated from Streptomyces, can bind with the 60S ribosomal subunit and prevent peptide bond formation to result in block of peptide elongation and degradation of polyribosome, functionally inhibiting synthesis of numerous proteins and DNA [18]. Our previous studies show that anisomycin can significantly suppress cancer cell growth in vitro [19]. However, high cytotoxicity against normal cells limits the improvement of anticancer efficacy of anisomycin. In order to achieve enhanced anticancer efficacy and low cytotoxicity against normal cells, we prepared functionalized selenium nanoparticles by binding anisomycin with the surface of SeNPs. It has been found that particle size can affect the effectiveness of cellular uptake [20]. Herein, tremendous efforts have been made in tailoring the size of functionalized selenium nanoparticles SeNPs@Am by adjusting the reaction concentrations of sodium selenite or anisomycin, which leads to a series of SeNPs@Am with size ranging from 56 to 185 nm. The SeNPs@Am presents good dispersibility, stability, and superior biocompatibility—all of which are crucial for biomedical applications. To the best of our knowledge, no study on the correlation between selenium nanoparticle size and cellular uptake effectiveness has been reported so far. Thus, we investigated the effect of SeNPs@Am size on cellular uptake of HepG2 cells. The data from cellular uptake shows that the maximum uptake by HepG2 cells occurs at a nanoparticle size of 56 nm. This result will have implications in designing selenium nanoparticles optimized as anticancer drug carriers. SeNPs@Am can effectively induce the HepG2 cell apoptosis and preclude the migration of HepG2 cells, and possess great selectivity between HepG2 cells and normal cells. The underlying action mechanisms of SeNPs@Am were further investigated in detail. Taken together, our results suggest that SeNPs@Am can be an ideal nanodrug for hepatocellular carcinoma.

## Methods

### Materials

Anisomycin, sodium selenite (Na_2_SeO_3_), thiazolylbluetetrazolium bromide (MTT), and 4′,6-diamidino-2-phenylindole (DAPI), which were of analytical or biological reagent grade without further purification, were purchased from Sigma. Propidium iodide (PI) and Annexin V-FITC Kit containing PI were purchased from KeyGen Biotech, China. Ascorbic acid (Vc) was bought from a Guangzhou chemical reagent factory. Water used in all experiments was produced by a Milli-Q water purification system (Millipore).

### Synthesis of SeNPs with Various Sizes

Na_2_SeO_3_ powder and anisomycin were dissolved in super-purified water to prepare 5 mM Na_2_SeO_3_ stock solution and 20 mM anisomycin solution, respectively. Aqueous solution containing 20 mM Vc was freshly made for every experiment. SeNPs with various sizes were synthesized according to the methods in the literature with minor modifications. Briefly, 0.0625, 0.125, 0.25, 0.5, and 1 mL of Vc solution were dropwise added to Na_2_SeO_3_ solution (1:1, *v*/*v*), and the mixture was reconstituted to a final volume of 2.5 mL with Milli-Q water. Then, the mixed solution was stirred for 12 h at 25 °C, and the final concentration of Na_2_SeO_3_ was 0.125, 0.25, 0.5, 1.0, and 2.0 mM, respectively. Excess Na_2_SeO_3_ and Vc were removed by dialysis against Milli-Q water overnight. The pure SeNPs with various sizes were obtained.

### Synthesis of SeNPs@Am with Various Sizes

To prepare Am-Vc mixed solution, 12.5, 25, 50, 100, and 200 μL of Am solution were mixed with 125 μL Vc solution, respectively. The Am-Vc mixed solution was dropwise added to 0.125 mL Na_2_SO_3_ solution, and the mixture was reconstituted to a final volume of 2.5 mL with Milli-Q water. Then, the mixed solution was stirred for 12 h at 25 °C, and the final concentration of anisomycin was 0.1, 0.25, 0.5, 1.0, and 2.0 mM. The prepared nanoparticles SeNPs@Am were purified by dialysis against super-purified water for 12 h. The pure SeNPs@Am of 67, 56, 75, 122, and 185 nm in size were obtained. Finally, the solution was subjected to centrifugation at 10,000*g* for 2 h and freeze-dried. SeNPs@Am powder was stored at −20 °C until use. The SeNPs@Am of 56 nm in size was applied for further biological studies. Inductively coupled plasma mass spectrometry (ICP-MS) was applied for determination of Se concentration. To examine intracellular uptake and localization of SeNPs@Am in HepG2 cells, it was labeled with 10 μg of coumarin-6, a fluorescent dye, through the above-described procedure after addition of Vc solution.

Various methods were used to characterize properties of the prepared nanoparticles. Briefly, transmission electron microscopy (TEM) samples were prepared by adding the nanoparticles collosol onto a holey carbon film on copper grids. The TEM images were obtained on Hitachi (H-7650) at an accelerating voltage at 80 kV. Energy dispersive X-ray spectroscope (EDS) was used on an EX-250 system (Horiba) to test elemental composition of the SeNPs@Am. Fourier transform infrared spectrometry (FTIR) analysis for all samples was carried out on an Equinox 55 IR spectrometer. Size distribution and zeta potential of SeNPs@Am nanoparticles were examined by photon correlation spectroscopy (PCS) on a Nano-ZS instrument (Malvern Instruments Limited). X-ray photoelectron spectroscopy (XPS) measurement was completed on an ESCALAB 250 spectrometer with the monochromatic Al Kα X-ray radiation (energy 1.49 keV, 500 μm spot size).

### Cell Line and Cell Culture

HepG2 and HUVEC-12 cell lines were offered by American Type Culture Collection (Manassas, VA) and cultured in RPMI-1640 medium containing 10 % fetal bovine serum (FBS), 100 units/mL of penicillin, and 50 units/mL of streptomycin at 37 °C in an incubator containing 5 % CO_2_.

### In vitro Cellular Uptake and Living Cell Imaging of SeNPs@Am

Intracellular uptake of SeNPs@Am was qualitatively analyzed as previously described [21]. Briefly, HepG2 cells were incubated in 6-well plates (80,000 cells/well) at 37 °C for 24 h. The medium in the well was replaced with fresh medium containing different concentrations of the coumarin-6 loaded SeNPs@Am (at the actual concentrations of Se) and incubated for 2 h at 37 °C in a CO_2_ incubator. At the end of the incubation, the cells were washed three times with cold phosphate buffered saline (PBS). Then, the cells were stained with 5 μg/mL of DAPI for 20 min. After that, the cells were washed three times with cold PBS, and the intracellular uptake imaging of SeNPs@Am was observed under a fluorescent microscope (Nikon Eclipse 80i). The living cell imaging of SeNPs@Am was observed using the similar method mentioned above. For quantitative analysis of cellular uptake, Se concentrations in the cells after the treatment were determined by the ICP-MS method. Briefly, the HepG2 and HUVEC-12 cells were incubated with fresh medium containing different concentrations of the SeNPs@Am (at the actual concentrations of Se) for various times at 37 °C in a CO_2_ incubator. Then, the cells were washed with PBS three times and were lysed after adding 0.2 M NaOH solution containing 0.5 % Triton X-100. The product was reconstituted to 1 mL with Milli-Q H_2_O and used for ICP-MS analysis. Colocalization of coumarin-6-loaded SeNPs@Am in HepG2 cells was carried out by separately staining with the lysosomal marker, Lyso Tracker Red-DND-99 (Sigma-Aldrich Corporation), and nuclear marker DAPI (Sigma-Aldrich Corporation). Briefly, the cells were cultured in 6-well plates to 70 % confluence and washed with cold PBS. Then, they were separately incubated with fresh complete medium containing Lyso Tracker, DAPI, and 25 μM of the 6-coumarin-loaded SeNPs@Am (at the actual concentrations of Se) at 37 °C in 5 % CO_2_ for different times, respectively. Then, the stained cells were observed under a fluorescence microscope (TE2000-S).

### In vitro Drug Release

In a hard glass tube with continuous shaking at 37 °C, 5 mg of SeNPs@Am powder was dissolved in 5 mL PBS (pH 7.4 and 5.4). At different time intervals, a specific slight amount of PBS was replaced by an equivalent volume of PBS. Concentrations of anisomycin were analyzed using a HPLC system (Agilent 1100) equipped with μ-Bondapak C_18_ (4 × 300 mm) column, and a detection wavelength was set at 225 nm. Mobile phase is made by mixing 125 mL of acetonitrile with 875 mL of 0.05 M potassium dihydrogen phosphate buffer solution (pH 6.0) in a 1-L vacuum flask, and flow rate was set at 1.0 mL/min.

### Cellular Uptake Pathway of SeNPs@Am

HepG2 cells were seeded in a 6-well plate at a density of 2 × 10^5^ cells/well and cultured in an incubator with 5 % CO_2_ atmosphere. After 24 h, the cells were washed once with PBS and preincubated in serum-free medium for 1 h with several endocytic inhibitors: 3 mg/mL of NaN_3_/50 mM of 2-deoxy-d-glucose (DOG), 2 μg/mL of colchicine, 50 μg/mL of monensin, and 0.45 M of sucrose. After 1 h of incubation, the medium was replaced with fresh medium containing 25 μM of SeNPs@Am and further incubated at 37 °C in 5 % CO_2_ for 1 h. Then, the cells were washed with PBS three times and were lysed after adding 0.2 M NaOH solution containing 0.5 % Triton X-100. The cells treated with only SeNPs@Am (no inhibitor) were used as positive controls. To determine concentrations of Se, all the samples would be collected for ICP-MS analysis. Additionally, the cells were cultured in the medium containing 10 mM NaN_3_/50 mM DOG or the complete medium containing SeNPs@Am at 4 °C for 4 h to analyze whether it is energy-dependent. Uptake (%) was calculated based on the following equation:

$$ \begin{array}{c}\mathrm{Uptake}\ \mathrm{of}\ \mathrm{SeNPs}@\mathrm{Am}\ \left(\%\right) = \Big(\mathrm{uptake}\ \mathrm{of}\ \mathrm{SeNPs}@\mathrm{Am}\ \mathrm{in}\ \mathrm{presence}\ \mathrm{of}\ \mathrm{in}\mathrm{hibitor}/\ \mathrm{uptake}\ \mathrm{of}\ \\ {}\mathrm{SeNPs}@\mathrm{Am}\ \mathrm{in}\ \mathrm{absence}\ \mathrm{of}\ \mathrm{in}\ \mathrm{hibitor}\mathrm{s}\Big) \times 100\end{array} $$

### Cell Viability Assay

Cell proliferation inhibition was tested by a MTT assay. HepG2 and HUVEC-12 cells were seeded in 96-well plates at a density of 6 × 10^3^ and 2 × 10^3^ cells/well at 37 °C in 5 % CO_2_ for 24 h, respectively. The cells were exposed to 0.2 mL fresh medium containing SeNPs@Am (in an equivalent anisomycin concentration level), anisomycin or SeNPs at different concentrations for 48 h. After that, the previous culture medium was removed and washed with PBS twice. Then, 20 μL of 5 mg mL^−1^ MTT solution and 180 μL fresh medium were added to each well and incubated at 37 °C in 5 % CO_2_ for 4 h. The medium with MTT was discarded before 150 μL of DMSO was added to each well to dissolve the formazan crystals. An absorbing value of each well at 490 nm was analyzed by a 680-type microplate reader (Bio-Rad, Berkeley, CA, USA). Results are expressed as percentage of MTT reduction relative to absorbance of control cells [22].

### Wound-Healing Assay

HepG2 cells were seeded at a density of 1 × 10^5^ cells/well on 24-well plates and incubated to 100 % confluence. The adherent monolayer cells were scratched by using a micropipette tip and washed twice with PBS to remove suspended cells. The cells were exposed to SeNPs@Am (0.2 μM, in an equivalent anisomycin concentration level), anisomycin (0.2 μM), or SeNPs (2 μM) at 37 °C in 5 % CO_2_. After 6 h, the medium was replaced with fresh RPMI-1640 with 2 % FBS. The serial images of scratched monolayer cells were captured at 0 and 24 h by an inverted microscope. Average scratch width was determined at three random areas, and migration rate was calculated as follows.

$$ \mathrm{Cell}\ \mathrm{motility}\ \left(\%\right) = \left[1-\left(\mathrm{distance}\ \mathrm{of}\ \mathrm{the}\ \mathrm{wound}\ \mathrm{at}\ 24\ \mathrm{h}/\ \mathrm{distance}\ \mathrm{of}\ \mathrm{the}\ \mathrm{wound}\ \mathrm{at}\ 0\ \mathrm{h}\right)\right] \times 100\kern0.1em \%. $$

### Transwell Migration Assay

Ability of HepG2 cells to migrate was assessed by transwell-chamber (BD Biosciences, pore size, 8 μm) migration assay. Briefly, the cells were treated with SeNPs@Am (0.2 μM, in an equivalent anisomycin concentration level), anisomycin (0.2 μM), or SeNPs (2 μM) for 24 h, respectively. Then, the cells at a density of 1 × 10^5^ cells/mL were re-suspended in 100 μL serum-free medium and added to the upper chamber, whereas 400 μL medium containing 10 % FBS was applied to the lower chamber. The cells were next incubated at 37 °C in 5 % CO_2_ for 24 h, filter inserts were removed from the wells, and the cells in the upper chamber were wiped with a cotton swab. The cells in the lower chamber were fixed with methanol for 10 min and stained with eosin dye for 1 min at room temperature. Thereafter, the migrating cells in five fields were randomly captured and counted under a light microscope. Results are expressed as the migration cells in the experimental group relative to those in the control group. Inhibition rate of cell migration was calculated according to the equation, in which Mig_ctrl_ is from the control cells that migrate into the lower surface and Mig_t_ is from the treated cells that migrate into the lower surface.

$$ \mathrm{Inhibition}\ \mathrm{of}\ \mathrm{migration}\ \left(\%\right) = \left(\mathrm{M}\mathrm{i}{\mathrm{g}}_{\mathrm{ctrl}} - \mathrm{M}\mathrm{i}{\mathrm{g}}_{\mathrm{t}}\right)/\ \mathrm{M}\mathrm{i}{\mathrm{g}}_{\mathrm{ctrl}} \times 100\ \% $$

### Cell Cycle Analysis

For analysis of cell cycle distribution, the cells were exposed to 0.05, 0.1, and 0.2 μM of SeNPs@Am (in an equivalent anisomycin concentration level) at 37 °C in 5 % CO_2_ for 24 h and harvested by centrifugation. The harvested cells were washed with cold PBS and fixed in cold 70 % ethanol at −20 °C overnight. Then, the cells were washed with cold PBS and incubated with 0.1 mg/mL RNase, 20 μg/mL PI, and 0.1 % Triton X-100. DNA content of the cells was analyzed by using a FACSCalibur flow cytometer with a CellQuest software (Becton Dickinson, USA).

### Annexin V-FITC/PI Staining

To evaluate extent of cell apoptosis, the HepG2 cells treated with 0.05, 0.1, and 0.2 μM of SeNPs@Am (in an equivalent anisomycin concentration level) were harvested and washed with cold PBS twice. The cells were re-suspended in 100 μL diluted binding buffer solution and stained using an Annexin V-FITC Kit containing PI (KeyGen Biotech, China). They were kept at room temperature in darkness for 15 min. Before the detection, 200 μL of diluted binding buffer was added. Finally, apoptotic proportion of the treated cells was measured using flow cytometry (FACSCalibur, Becton Dickinson).

### TUNEL-DAPI Staining Assay

Apoptotic DNA fragmentation was detected by a TUNEL assay according to manufacturers’ protocol. Briefly, HepG2 cells were treated with 0.1 and 0.2 μM of SeNPs@Am (in an equivalent anisomycin concentration level) for 24 h. They were fixed with 4 % formaldehyde for 10 min and washed with PBS before permeabilization with PBS containing 0.1 % Triton X-100. Then, TUNEL reaction mixture was added to the cells at room temperature for 1 h. The cell nuclei were stained with 1 μg mL^−1^ DAPI for 15 min before the end of the TUNEL staining. Finally, the stained cells were photographed under a fluorescence microscope (TE2000-S).

### Western Blot Analysis

After being treated with 0.2 μM SeNPs@Am (in an equivalent anisomycin concentration level), 0.2 μM anisomycin, or 2 μM SeNPs, the HepG2 cells were washed and lysed by a RIPA Lysis Kit (Beyotime Institute of Biotechnology, China) containing phenylmethylsulfonyl fluoride (PMSF). The protein concentration of cytosolic extract was measured with a BCA Protein Assay Kit (Beyotime). An equal amount of the protein was separated by SDS-PAGE and then transferred onto nitrocellulose membranes (Amersham Biosciences, Pittsburgh, PA, USA). The membranes were blocked with 5 % non-fat milk at room temperature for 1 h and then washed three times with tris buffered saline (TBS) containing 0.05 % Tween 20 for 5 min each time. Thereafter, the membranes were probed at 4 °C overnight with primary antibodies, respectively, that included anti-ICBP90, anti-p16, anti-p21, anti-P-p21(Thr145), anti-p27, anti-P-p27 (Ser10), anti-P-CDK2 (Thr160), anti-p53, anti-P-p53(Ser20), anti-E2F1, anti-p73, anti-P-p73 (Tyr99), anti-Rb, anti-P-Rb (Ser807), and anti-β-actin. Then, the membranes continued to be incubated with relative second antibodies at room temperature for 1 h. The bands were visualized by enhanced chemiluminescence (Cell Signaling Technology, Inc. USA) according to the manufacturer’s instruction. The band density was checked by a FluorChem 8000 system (Alpha Innotech, Santa Clara, CA, USA).

### Statistical Analyses

Statistical analyses were performed with SPSS 17.0 software (SPSS Inc., IL, US). The results were expressed as the means ± SD of three independent experiments. Individual comparisons were made by one-way ANOVA for multiple comparison data, and *p* values less than 0.05 were considered to be statistically different and *p* values less than 0.01 to be significantly different.

## Results and Discussion

### Physical and Chemical Characterization of SeNPs@Am

**Fig. 1:**
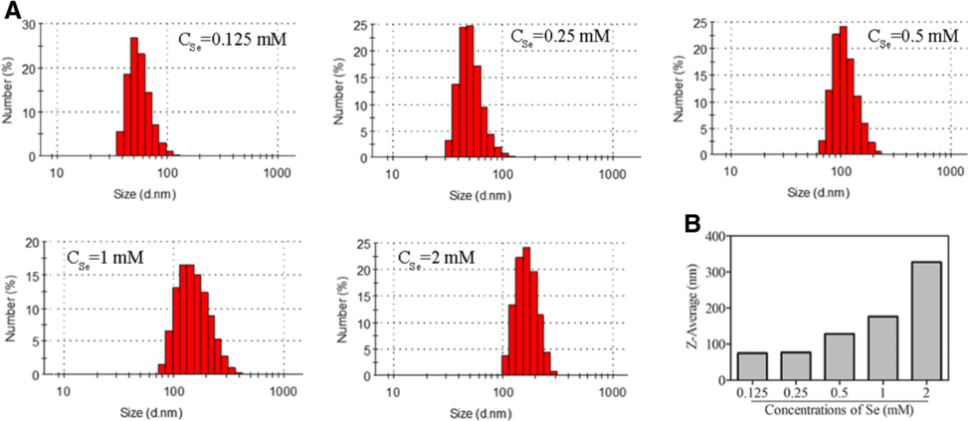
**a** Distribution of SeNPs with various size at different reaction concentrations of Se, respectively. **b** The average diameter of above SeNPs, respectively

**Fig. 2:**
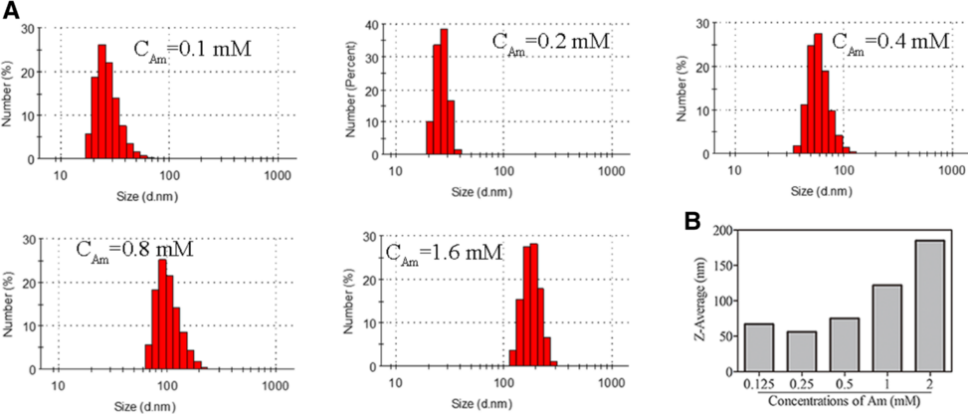
**a** Distribution of SeNPs@Am with various size at different reaction concentrations of anisomycin, respectively. **b** The average diameter of above SeNPs@Am, respectively

**Fig. 3:**
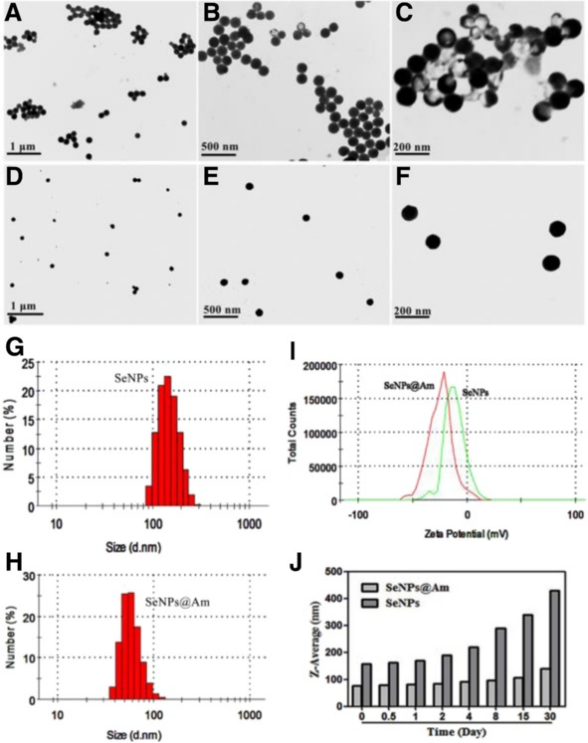
Characterization of SeNPs and SeNPs@Am. **a**–**c**, **d**–**f** TEM images of SeNPs and SeNPs@Am, respectively. **g**, **h** Particle size and distribution of SeNPs and SeNPs@Am, respectively. **i** Zeta potential of SeNPs and SeNPs@Am. **j** Particle size growth of SeNPs and SeNPs@Am during 30 days

The particle size impacts on all applications of nanoparticles in biomedicine. Thus, it is very meaningful to control the size of nanomaterials. It is known that type of materials, the ratio of reaction substrates, and their final concentrations influence the size, size distribution, and chemical composition of particles. In this study, the functionalized selenium nanoparticle SeNPs@Am with various sizes were synthesized through adjusting the reaction concentrations of sodium selenite or anisomycin. As shown in Fig. 1, an increase in particle size was observed when the reaction concentrations of sodium selenite was increased from 0.125 to 2 mM. Here, 0.5 mM was chosen as the optimized reaction concentration of sodium selenite. Figure 2 presents the size graph of SeNPs@Am synthesized at the different reaction concentrations of anisomycin ranging from 0.1 to1.6 mM. The presence of 0.1~0.4 mM anisomycin significantly decreased the particle size to 67, 56, and 75 nm, respectively. However, with increasing the concentration of anisomycin up to 1.6 mM, the size of SeNPs@Am dramatically increased to 185 nm. These datum indicate that the sizes of functionalized selenium nanoparticle SeNPs@Am can be regulated by adjusting the reaction concentrations of sodium selenite and anisomycin. The SeNPs@Am of 56 nm in size was used as a preferred nanoscale drug for its suitable size. TEM images of the prepared SeNPs (Fig. 3a–c) and SeNPs@Am (Fig. 3d–f) clearly revealed that SeNPs modified with anisomycin presented a homogeneous and monodisperse spherical structure with the diameter of approximately 60 nm. In contrast, SeNPs without anisomycin easily aggregated owing to the high surface energy of SeNPs, and precipitated in the aqueous solution with an average diameter of approximately 110 nm. Particle size, distribution, and zeta potential of SeNPs@Am were measured to examine effects of anisomycin on stability and surface properties of SeNPs. Our data indicated that the presence of anisomycin dramatically decreased the average diameter of SeNPs from 125 to 63 nm (Fig. 3g, h). After surface modification with anisomycin, the zeta potential of particles was obviously decreased from −11.5 to −24.4 mV, suggesting that the SeNPs@Am exhibited higher stability than SeNPs (Fig. 3i). In addition, we found that SeNPs@Am kept stable during 8 days in water solution. In contrast, the particle size of SeNPs alone dramatically increased up to ~300 nm after 8 days (Fig. 3j).

**Fig. 4:**
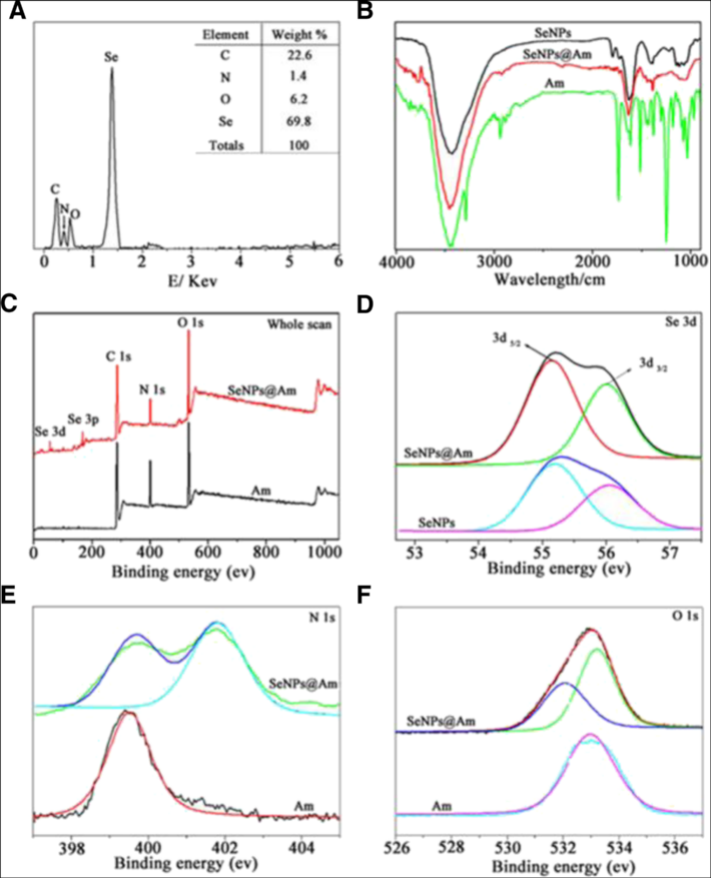
Chemical composition and structure characterization of SeNPs@Am. **a** EDS analysis of SeNPs@Am. **b** FTIR spectra of SeNPs, SeNPs@Am and Am. **c** XPS spectra of SeNPs@Am and Am. **d** Se 3d spectra of SeNPs@Am and Am. **e** N 1 s spectra of SeNPs@Am and Am. **f** O 1 s spectra of SeNPs@Am and Am

EDS analysis showed presence of a signal from Se atom (69.8 %), together with N (1.4 %), C (22.6 %), and O (6.2 %) atoms from anisomycin molecules. The presence of an N signal peak confirmed that SeNPs were successfully conjugated with anisomycin (Fig. 4a). Based on the results from EDS, the representative chemical formula for SeNPs@Am is derived as (Se_9_Am)n. FTIR spectroscopy was further used to find out whether there was a formation of chemical bonds between anisomycin and Se. In the spectrum of anisomycin, the peaks at 2938 and 1376 cm^−1^ were attributed to the stretching vibrations of C−C and C−N, respectively. The appearance of the above peaks in the spectrum of SeNPs@Am suggested the presence of anisomycin on the surface of SeNPs (Fig. 4b). XPS was also recorded for interaction between SeNPs@Am and anisomycin. The N 1 s peak at about 400 eV in the spectrum of SeNPs@Am showed that anisomycin was conjugated to the SeNPs (Fig. 4c). The peaks of Se 3d_5/2_ and 3d_3/2_ also shifted from 55.2 and 56.05 eV (SeNPs) to 55.15 and 56.0 eV (SeNPs@Am), respectively, suggesting a strong interaction between anisomycin and Se nanoparticles (Fig. 4d). Meanwhile, the spectrum of N 1 s and O 1 s peaks in SeNPs@Am both split into two, respectively (Fig. 4e, f). Therefore, these results further confirmed the formation of Se–N and Se–O bonds in SeNPs@Am.

### Enhanced Cellular Uptake of SeNPs@Am

**Fig. 5:**
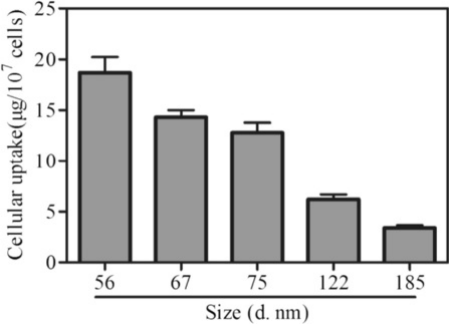
Size-dependent cellular uptake effciency of SeNPs@Am by HepG2 cells. Values expressed are means ± SD of triplicate

Intracellular uptake of nanomaterial-based drugs is a key factor that usually contributes to drug cytotoxicity [23]. To investigate the size effect on cellular uptake, the uptake of SeNPs@Am with various sizes by HepG2 cells was examined. As shown in Fig. 5, cellular uptake is particle-size-dependent ranging from 56 to 185 nm and the maximum uptake by HepG2 cells occurs at a nanoparticle size of 56 nm, suggesting that the size of particles plays a very important role in the cellular uptake. It has been reported that nanoparticles with the diameter of ~55 nm have the fastest wrapping time and the receptor-ligand interaction can produce enough free energy to drive the nanoparticles into the tumor cells. This minimum wrapping time led to more accumulation of the ~55-nm nanoparticles into the tumor cells [24].

**Fig. 6:**
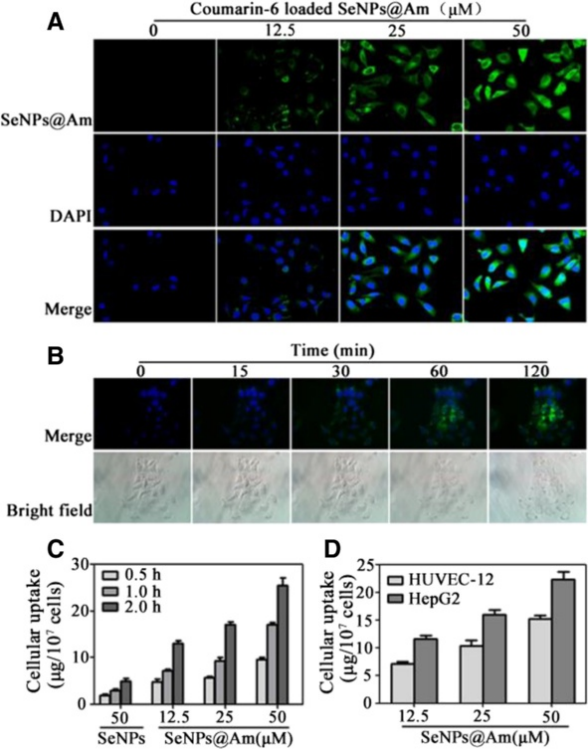
Cellular uptake of SeNPs@Am. **a** Fluorescence microscope images show the internalization of coumarin-6-loaded SeNPs@Am (*green fluorescence*) in HepG2 cells. **b** Real-time imaging for HepG2 cells treated with coumarin-6-loaded SeNPs@Am. The *upper panel* is merged images of the nanoparticles and nuclei, and the *lower panel* is DIC images. Magnification, ×400. **c** Time- and dose-dependent cellular uptake efficiency of SeNPs@Am by HepG2 cells. **d** Dose-dependent cellular uptake efficiency of SeNPs@Am by HUVEC-12 and HepG2 cells. Values expressed are means ± SD of triplicate

To investigate cellular uptake effectiveness in HepG2 cells, a short-term particle endocytosis test was visually carried out using coumarin-6-loaded SeNPs@Am. Green fluorescence from coumarin-6-loaded SeNPs@Am penetrating into HepG2 cells was enhanced in a dose-dependent manner, following incubation of the cells with the labeled SeNPs@Am for 4 h (Fig. 6a). Consistent with other reports, SeNPs@Am mainly accumulated in cytoplasm, but was not detected in the nucleus, indicating that the nuclei were not the cellular target of SeNPs@Am [25]. The intracellular SeNPs@Am increased in a time-dependent manner. Cellular uptake of SeNPs@Am appeared at 15 min, and then the intracellular SeNPs@Am gradually increased during 2 h treatment (Fig. 6b). It was worth mentioning that the cells treated with SeNPs@Am exhibited markedly morphologic changes, where lots of cells became round and adherent cells tended to be detached. The morphological changes in HepG2 cells might be due to the cytotoxicity of cellular accumulation of SeNPs@Am.

A quantitative analysis of cellular uptake was conducted by ICP-MS [26]. Internalization of SeNPs and SeNPs@Am was investigated in HepG2 and HUVEC-12 cells, respectively. Intracellular SeNPs@Am concentrations were increased in HepG2 cells in a time- or dose-dependent manner (Fig. 6c). As shown clearly in Fig. 6d, the cellular uptake ability in HepG2 cells was greater in comparison with that in HUVEC-12 cells. The higher cellular uptake of SeNPs@Am in HepG2 cells may be due to its favorable membrane permeability.

The above datum indicate that nanoparticles SeNPs@Am with 56 nm in size are well uptaken by HepG2 cells and are the most suitable candidates for further studies in biological application.

### Localization, Uptake Channel, and Release of SeNPs@Am

**Fig. 7:**
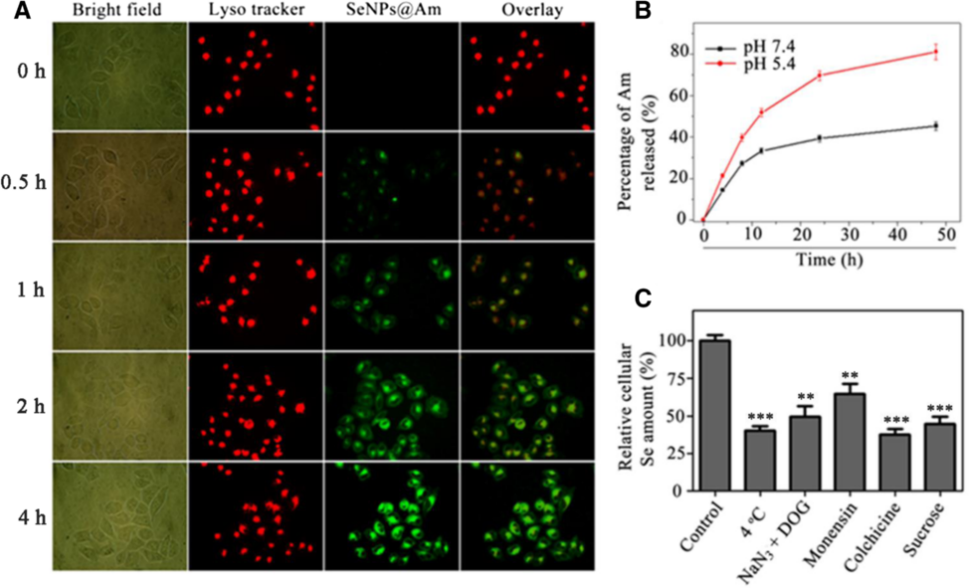
Colocalization of SeNPs@Am and lysosomes in HepG2 cells. **a** HepG2 cells were treated with lysosomal marker lyso tracker red (*red fluorescence*) and coumarin-6-loaded SeNPs@Am (*green fluorescence*) at 37 oC for different time and visualized under a fluorescence microscope (magnification, ×400). **b** In vitro release profile of Am from SeNPs@Am in RPMI-1640 medium with 10 % fetal bovine serum. Am concentrations were determined by HPLC analysis. **c** Cellular amount of Se in HepG2 cells after 4 h of incubation with SeNPs@Am. The cells were incubated for 4 h either at 37 °C (control) or at 4 °C. Prior to the incubation with SeNPs@Am, the cells were pretreated with specific endocytosis inhibitors for 1 h. **p < 0.01, ***p < 0.001 vs. the control

Intracellular localization of SeNPs@Am was explored by lyso tracker red and DAPI for staining of lysosome and nucleus, respectively [27]. The merged images clearly showed that most of SeNPs@Am resided in the lysosomes, followed by a gradual dosage increasement during 4 h of treatment (Fig. 7a). This result verifies that lysosome is a main organelle target for SeNPs@Am.

Controlled and sustained drug release is very important for drug delivery systems [28]. Generally speaking, pH value in tumor tissue is lower than normal, which is attributed to lactic acid produced due to hypoxia and acidic intracellular organelles. Thus, we carried out drug release kinetic measurement at both pH 7.4 and 5.4 to mimic physiological and lysosomal pH (Fig. 7b). Anisomycin release from SeNPs@Am was much lower at pH 7.4 (45.4 %) than at pH 5.4 (81.0 %) for 48 h. At lower pH, more anisomycin molecules were protonated, which resulted in a weaker binding force between anisomycin and SeNPs. This initial rapid release of anisomycin can be partly due to the adsorption of drug on the surface of nanoparticles.

Endocytosis is one of the most important entry mechanisms for nanoparticles [29]. In living cells, the endocytosis involves three major pathways, including caveolae-mediated endocytosis, macropinocytosis, and clathrin-mediated endocytosis. Several specific endocytosis inhibitors, such as monensin, colchicine, and sucrose, were used to elucidate cellular uptake channels and endocytosis mechanisms of SeNPs@Am in HepG2 cells. Treatments with NaN_3_ and DOG, or at 4 °C, dramatically decreased the cellular uptake of SeNPs@Am, demonstrating that SeNPs@Am enters HepG2 cells via energy-dependent endocytosis. Cellular uptake of SeNPs@Am was decreased markedly by colchicine and sucrose endocytosis inhibitors, indicating that SeNPs@Am enters the cells via macropinocytosis and/or clathrin-mediated endocytosis pathways (Fig. 7c).

### In vitro Cytotoxicity of SeNPs@Am

**Fig. 8:**
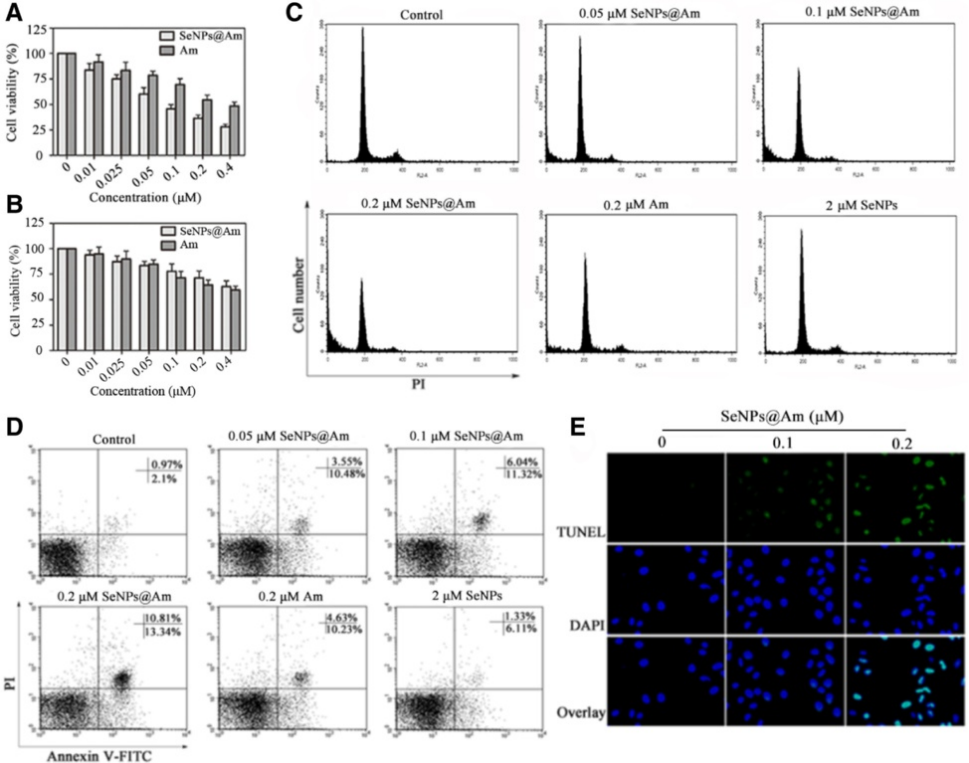
SeNPs@Am alters HepG2 cell biobehaviors via affecting the cell proliferation, cycle and apoptosis. **a**, **b** Cell viability in HepG2 and HUVEC-12 cells were determined by the MTT assay after their exposure to SeNPs@Am and Am. **c** Cell cycle distribution was analyzed by flow cytometry. **d** The apoptotic proportion of cells was analyzed by flow cytometry. **e** Representative photomicrographs of DNA fragmentation and nuclear condensation induced by SeNPs@Am were detected by TUNEL-DAPI co-staining assay at 24 h post the treatment. Amplification, ×400

Cytotoxicity of SeNPs@Am against HepG2 or HUVEC-12 cells was investigated by MTT assay. Reducing cell survival to around 36.4 or 54.5 % (Fig. 8a), 0.2 μM of SeNPs@Am or anisomycin was enough to repress the viability of HepG2 cells in a dose-dependent manner. However, bare SeNPs as a carrier was slightly cytotoxic to HepG2 cells even at a dose of 16 μM (Additional file 1: Figure S1 in supporting information). The data indicates that SeNPs as a drug delivery system obviously enhances the anticancer activity of anisomycin on HepG2 cells. This can be explained by the fact that the controlled and sustained drug release of SeNPs@Am led to a considerably higher intracellular concentration of drug in HepG2 cells with a highly efficient anticancer activity. Meanwhile, SeNPs@Am showed a weak ability to kill HUVEC-12 (Fig. 8b). The result may be attributed to greater cellular uptake ability in HepG2 cells in comparison with HUVEC-12. The high activity of SeNPs@Am under low concentration supports its future medical applications.

### Influence of SeNPs@Am on Cell Cycle and Apoptosis

Flow cytometry was employed to study the impact of SeNPs@Am on cell cycle progression. HepG2 cells were stimulated with SeNPs@Am for 24 h and subjected to flow cytometry for cell cycle analysis [30]. The untreated cells were mainly in the G0/G1 phase, whereas the SeNPs@Am-treated cells cycled into the sub-G1 phase in a dose-dependent manner, suggesting that SeNPs@Am significantly induces HepG2 cell apoptosis. Meanwhile, SeNPs@Am resulted in increasement of the cells at the G0/G1 phase and decreasement at the S-phase and G2/M-phase with the increasement of the concentrations of SeNPs@Am. However, little change was observed in anisomycin or SeNPs groups (Fig. 8c).

To quantify apoptosis in HepG2 cells triggered by SeNPs@Am, the cells were analyzed by Annexin V-FITC and PI dual staining. The apoptotic rate of HepG2 cells was increased with the increasing dosage of SeNPs@Am, in which the early- and late-stage apoptotic rates of HepG2 cells treated with 0.2 μM of SeNPs@Am reached 10.81, and 13.34 %, respectively. However, the early- and late-stage apoptotic rates of HepG2 cells treated with 0.2 μM of anisomycin only reached 4.63 and 10.23 %, respectively (Fig. 8d). Therefore, SeNPs@Am exhibits greater ability to arrest the cell cycle and induce the apoptosis of HepG2 cells than anisomycin-free SeNPs or anisomycin alone. The SeNPs@Am-induced cell apoptosis was further determined by a TUNEL-DAPI assay [31]. HepG2 cells were exposed to 0.1 or 0.2 μM of SeNPs@Am for 24 h to display apoptotic properties, such as chromatin condensation, nuclear condensation, and formation of apoptotic bodies (Fig. 8e). These results support that SeNPs@Am represses HepG2 cell growth mainly through inhibiting the cell proliferation, arresting the cell cycle progression, and promoting the cell apoptosis.

### SeNPs@Am Precludes the Motility and Migration of HepG2 Cells

**Fig. 9:**
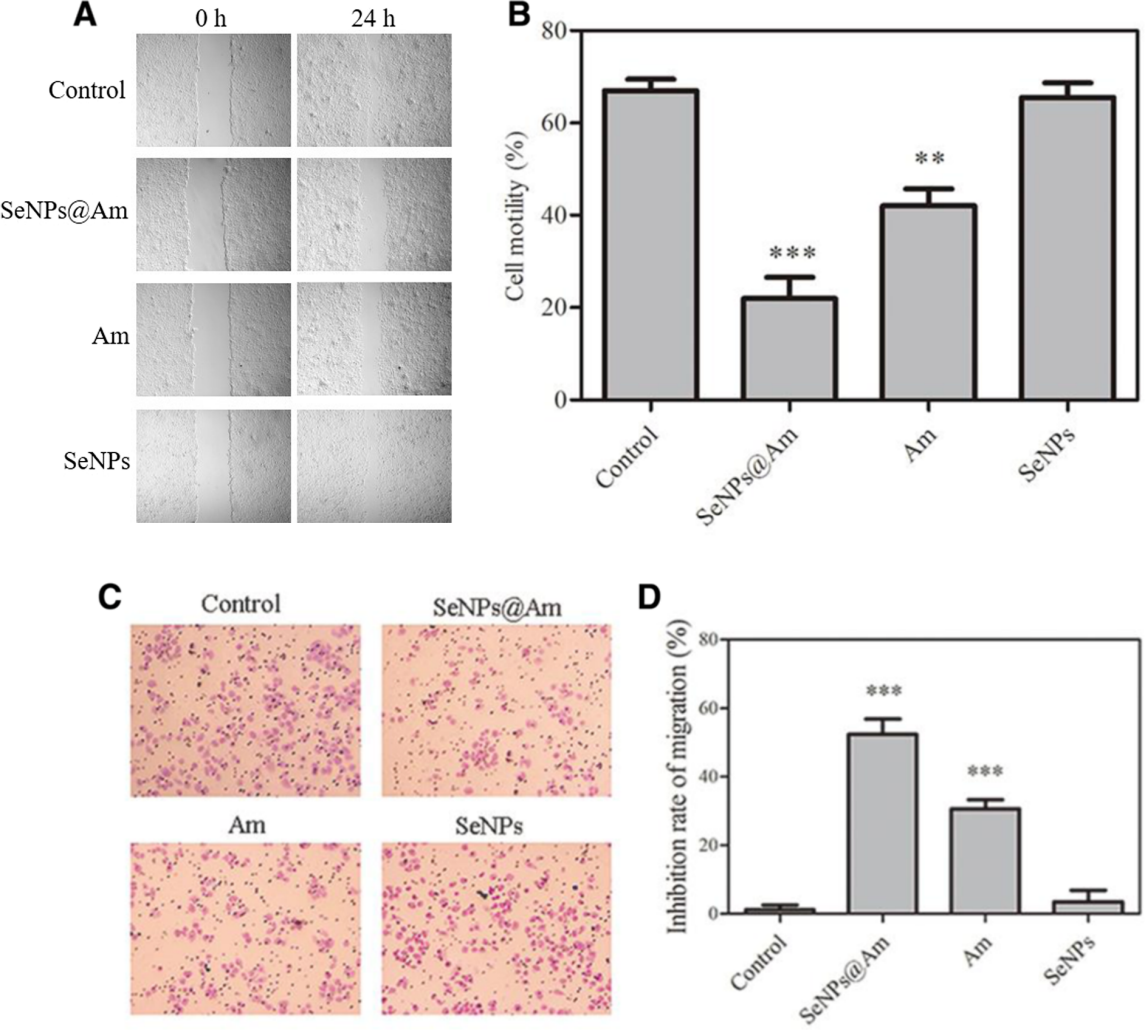
Wound edges were marked with lines. Amplification, ×100. **a** The wound-healing width was observed at the indicated time after the treatment of SeNPs@Am. **b** The cell motility of the control and treated HepG2 cells was quantitatively analyzed at 24 h. **c** The effect of SeNPs@Am, Am, and SeNPs on the migration of HepG2 cells. **d** The inhibition rate of migration of control, SeNPs@Am, Am, and SeNPs, respectively. Data represent the mean ± SD of three independent experiments. **p < 0.01, ***p < 0.001 vs. the control

A wound-healing assay was carried out to explore effect of SeNPs@Am, anisomycin, and SeNPs on HepG2 cell motility (Fig. 9a). Compared with the control, wound healing was obviously suppressed by SeNPs@Am or anisomycin, whereas SeNPs had negligible effect on cell migration. At 24 h, the wounds of the control, SeNPs@Am, and anisomycin groups were healed about 67.03 ± 1.7 %, 21.9 ± 3.1 %, and 42.7 ± 2.6 %, respectively (Fig. 9b). The results show that the HepG2 cell aggression was largely inhibited by SeNPs@Am, and SeNPs@Am is more effective than the free anisomycin.

A transwell filter assay was employed to further analyze tumor cell migration [32]. HepG2 cells can migrate across the pored filter membrane on the transwell [33]. At 24 h, the seeded cells migrated to the lower side of the filter membrane. SeNPs did not affect HepG2 cell migration. However, in the presence of SeNPs@Am or anisomycin, the cell migration was significantly inhibited, with the inhibition rate of 52.4 ± 3.4 % and 30.6 ± 2.5 %, respectively (Fig. 9c, d). The results of the transwell assay are concordant with the wound-healing assay, suggesting that SeNPs@Am is significantly superior to the free anisomycin to preclude the motility and migration of HepG2 cells.

### Effect of SeNPs@Am on the Expressions of Cell Cycle-Associated Proteins

**Fig. 10:**
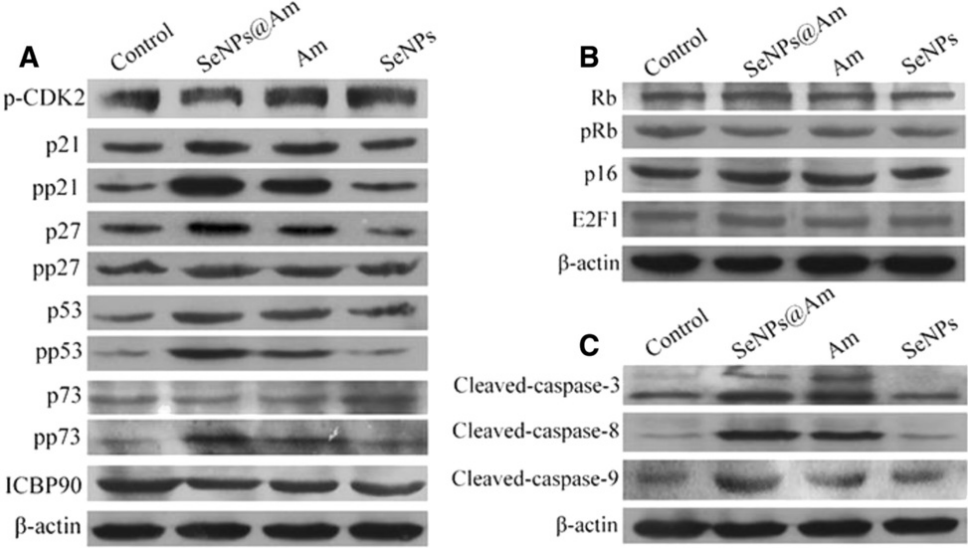
Effects of SeNPs@Am, Am, and SeNPs on the expression of cell cycle-related proteins. **a** p-CDK2, p21/pp21, p27/pp27, p53/pp53, p73/pp73, ICBP90. **b** Rb/pRb, p16, E2F1. **c** apoptosis-associated proteins cleaved-caspase-3, cleaved-caspase-8, and cleaved-caspase-9 in HepG2 cells

Cell cycle arrest is a major event to block tumor progression and metastasis. Cell cycle progression is mainly controlled by action of various types of cyclins and cyclin-dependent protein kinase (CDKs) [34]. Expressions of CDK inhibitors, such as p21, p27, and p53, regulate progression of cell cycle in G1 phase. As shown in Fig. 10a, the protein levels of phosphorylated CDK2 (an active form of CDK2) were down-regulated following treatment with 0.2 μM SeNPs@Am. On the other hand, the protein levels of p21/phosphorylated p21, p27/phosphorylated p27, and P53/phosphorylated p53 were significantly up-regulated. It is reported that the p21 cyclin-dependent kinase inhibitor gene can be activated by p73, and high-level expression of p21 can down-regulate ICBP90 through an ubiquitination-dependent protease degradation pathway [35, 36]. Fig. 10a shows that the expression of phosphorylated p73 was dramatically increased by SeNPs@Am, but the expression of the ICBP90 was decreased. These results indicate that SeNPs@Am causes cell cycle arrest through influencing expressions of the CDKs and related CDK inhibitors. The expression level of proteins from the SeNPs@Am-treated HepG2 cells were higher than that in the anisomycin-treated cells except p-CDK2 protein, suggesting SeNPs@Am exhibited greater activity than anisomycin to affect the protein expression. However, SeNPs as a drug carrier had little effects on protein expression.

A Rb pathway can suppress a transcriptional process of genes necessary for transition from G1- to S-phase. Phosphorylation of the Rb protein may be induced by cyclin D-CDK-4/6 complexes. On the contrary, its activation can be blocked by p16. The activation of the Rb protein facilitates the release of a transcription factor E2F1, leading to S-phase entry [37, 38]. As shown in Fig. 10b, Rb/phosphorylated Rb, p16, and E2F1 proteins in the SeNPs@Am-treated, anisomycin-treated, or SeNPs-treated HepG2 cells had little changes, suggesting that these proteins do not take part in the action of SeNPs@Am, anisomycin, or SeNPs on HepG2 cell biobehaviors.

### Impact of SeNPs@Am on the Expressions of Apoptosis-Associated Proteins

Caspase-3, caspase-8, and caspase-9 in caspase cascade signaling pathway are considered to be important proteases that can trigger cell apoptosis after cleaved [39]. Thus, we detected expressions of their activating forms, i.e., cleaved-caspase-3, cleaved-caspase-8, and cleaved-caspase-9 in the free treated, SeNPs@Am-treated, Am-treated, and SeNPs-treated HepG2 cells. SeNPs@Am can significantly induce the expressions of cleaved-caspase-3, cleaved-caspase-8, and cleaved-caspase-9 in HepG2 cells, and the activity of SeNPs@Am is stronger than that of anisomycin. SeNPs had little effects on them (Fig. 10c). Therefore, it is speculated that the activation of the cell cycle-regulating signals by SeNPs@Am may be connected to the activation of the caspase cascade signals.

## Conclusions

In summary, we have developed a drug delivery system based on selenium nanoparticles to successfully make a novel nanoparticle drug SeNPs@Am. It involves some key issues in the field of drug delivery. The SeNPs@Am with different sizes was prepared by adjusting the concentrations of reaction substrates. The SeNPs@Am of 56 nm in size presents the maximum cellular uptake in HepG2 cells. SeNPs@Am exhibits greater abilities to inhibit cell proliferation, arrest cell cycle, induce cell apoptosis, and block cell motility and migration than anisomycin does. Our results reveal that SeNPs@Am can inhibit human hepatocellular carcinoma multi-biobehaviors even in low concentration through activation of the P53/P73/P21/P27 signaling with inhibition of CDK-2 and ICBP90, and the caspase signaling, indicating that it may be a promising drug for hepatocellular carcinoma.

## Additional file

Additional file 1: Figure S1. Cell viability in HepG2 cells determined by the MTT assay after their exposure to SeNPs.

## Abbreviations

DAPI: 4′,6-diamidino-2-phenylindole
EDS: energy dispersive X-ray spectroscope
FBS: fetal bovine serum
FTIR: Fourier transform infrared spectrometry
HCC: hepatocellular carcinoma
ICP-MS: inductively coupled plasma mass spectrometry
MTT: thiazolylbluetetrazolium bromide
Na_2_SeO_3_: sodium selenite
NPs: nanoparticles
PCS: photon correlation spectroscopy
PI: propidium iodide
Se: selenium
SeNPs: Se nanoparticles
SeNPs@Am: anisomycin-loaded functionalized selenium nanoparticles
TEM: transmission electron microscopy
Vc: ascorbic acid
XPS: X-ray photoelectron spectroscopy
