# Correction to: Novel Functionalized Selenium Nanoparticles for Enhanced Anti-Hepatocarcinoma Activity In vitro

**DOI:** 10.1186/s11671-022-03700-9

**Published:** 2022-09-02

**Authors:** Yu Xia, Pengtao You, Fangfang Xu, Jing Liu, Feiyue Xing

**Affiliations:** 1 Department of Immunobiology, Institute of Tissue Transplantation and Immunology, Jinan University, Guangzhou, 510632 People’s Republic of China; 2 Department of Stomatology, Jinan University, Guangzhou, 510632 People’s Republic of China

## Correction to: Nanoscale Research Letters (2015) 10:349 https://doi.org/10.1186/s11671-015-1051-8

Following publication of the original article [1], the authors flagged that there was an error with the control subimage (24 h) in Fig. 9A of the article. The subimage in the control group (24 h) had been inadvertently duplicated from the SeNPs group. The subimage has since been replaced by the correct version, which can be seen below. Neither the legend of the figure nor the outcomes or conclusion of the article are affected by this correction.

**Fig. 9:**
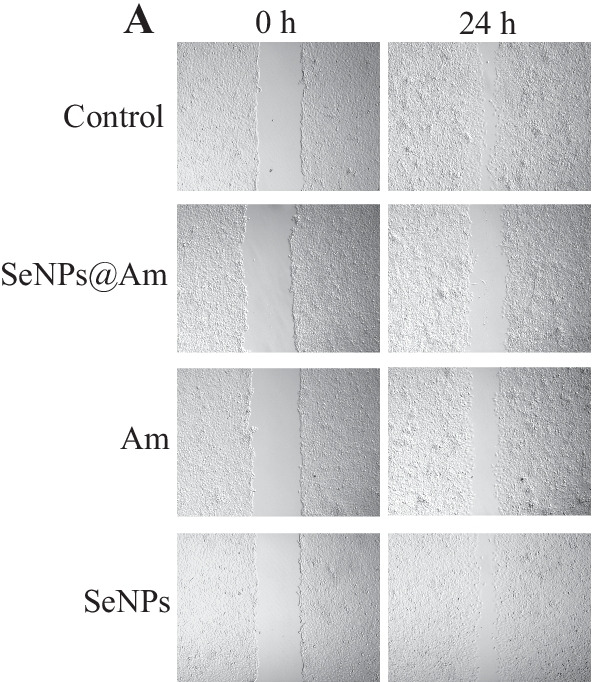
Wound edges were marked with lines. Amplification, ×100. **a** The wound-healing width was observed at the indicated time after the treatment of SeNPs@Am

The authors apologize for any inconvenience caused.
