# Supplementary material for: Novel Functionalized Selenium Nanoparticles for Enhanced Anti-Hepatocarcinoma Activity In vitro
Source: Nanoscale Res Lett. 2015 Sep 3;10:349. doi: 10.1186/s11671-015-1051-8 (PMC4558992; doi:10.1186/s11671-015-1051-8)
Supplement: Supplementary file 1 — Cell viability in HepG2 cells determined by the MTT assay after their exposure to SeNPs. [file 11671_2015_1051_MOESM1_ESM.docx]

Additional file 1

Supporting Information for

“Novel functionalized selenium nanoparticles for enhanced anti-hepatocarcinoma activity in vitro”

Yu Xia^1,2^, Pengtao You^1^, Fangfang Xu^1^, Jing Liu^3,^*, Feiyue Xing^1^^,^*

^1^Department of Immunobiology, Institute of Tissue Transplantation and Immunology, Jinan University, Guangzhou 510632, P.R. China; ^2^Department of Chemistry, Jinan University, Guangzhou 510632, P. R. China; ^3^Department of Stomatology, Jinan University, Guangzhou 510632, P.R. China

*Correspondence: Feiyue Xing or Jing Liu

Department of Immunobiology, Jinan University, Guangzhou 510632, P.R. China

Tel +86 20 85220723

Fax +86 20 85220723

Email : [tfyxing@jnu.edu.cn](mailto:tfyxing@jnu.edu.cn) or tjliu@jnu.edu.cn


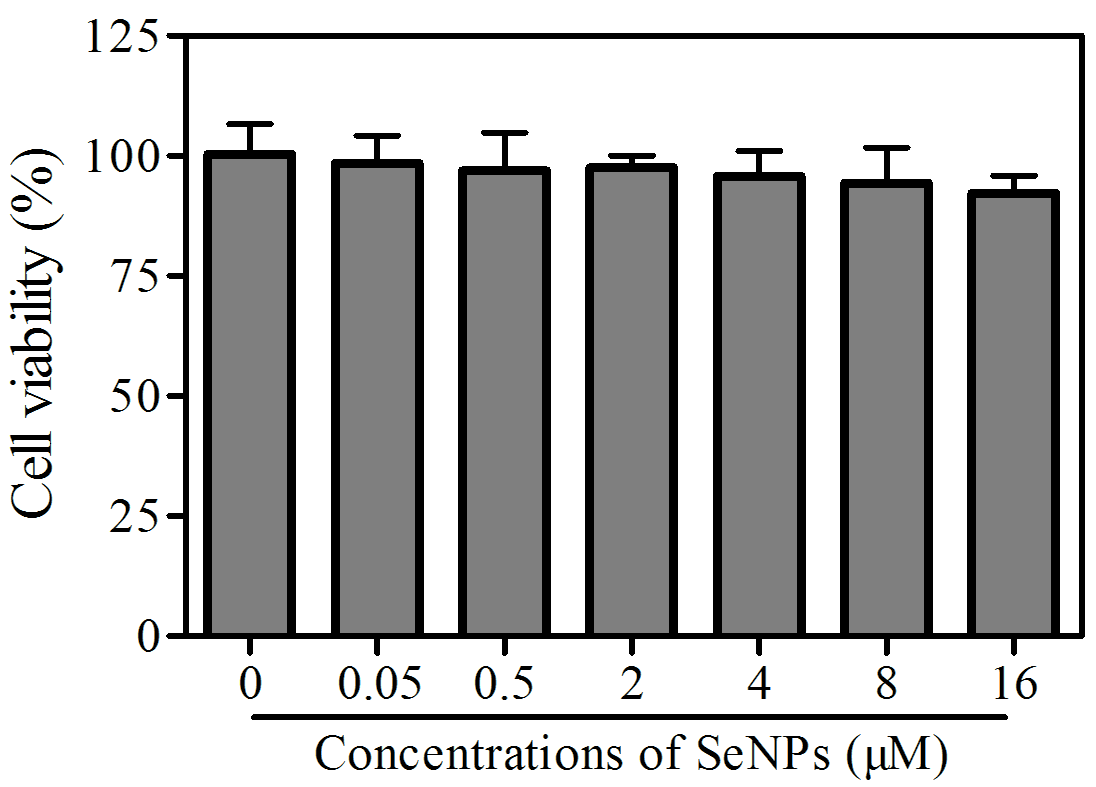


**Figure S1**  Cell viability in HepG2 cells were determined by the MTT assay after their exposure to SeNPs.
